# Rapid and Highly Controlled Generation of Monodisperse Multiple Emulsions via a One-Step Hybrid Microfluidic Device

**DOI:** 10.1038/s41598-019-49136-7

**Published:** 2019-09-03

**Authors:** Milad Azarmanesh, Saleh Bawazeer, Abdulmajeed A. Mohamad, Amir Sanati-Nezhad

**Affiliations:** 1 Department of Mechanical and Manufacturing Engineering, University of Calgary, Calgary, Alberta T2N 1N4 Canada; 2 Center for Bioengineering Research and Education, Biomedical Engineering Program, University of Calgary, Calgary, Alberta T2N 1N4 Canada

**Keywords:** Protein delivery, Mechanical engineering, Computational science

## Abstract

Multiple Emulsions (MEs) contain a drop laden with many micro-droplets. A single-step microfluidic-based synthesis process of MEs is presented to provide a rapid and controlled generation of monodisperse MEs. The design relies on the interaction of three immiscible fluids with each other in subsequent droplet formation steps to generate monodisperse ME constructs. The design is within a microchannel consists of two compartments of cross-junction and T-junction. The high shear stress at the cross-junction creates a stagnation point that splits the first immiscible phase to four jet streams each of which are sprayed to micrometer droplets surrounded by the second phase. The resulted structure is then supported by the third phase at the T-junction to generate and transport MEs. The ME formation within microfluidics is numerically simulated and the effects of several key parameters on properties of MEs are investigated. The dimensionless modeling of ME formation enables to change only one parameter at the time and analyze the sensitivity of the system to each parameter. The results demonstrate the capability of highly controlled and high-throughput MEs formation in a one-step synthesis process. The consecutive MEs are monodisperse in size which open avenues for the generation of controlled MEs for different applications.

## Introduction

**Figure 1:**
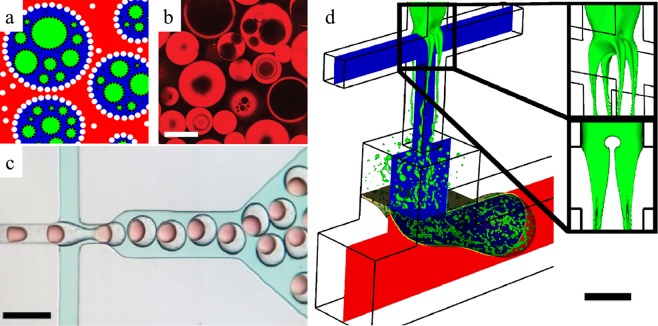
Formation of multiple emulsions (MEs) using different methods. (**a**) Red, blue, green, black and white are the Current phase, the Sheath phase, the Droplet phase and the emulsifiers, respectively. (**b**) MEs controlled by stimuli-responsive polymers. The scale bar is 30 µm. Reproduced from Besnard *et al*.^40^, with the permission of John Wiley & Sons, Ltd. Publishing. (**c**) The microfluidic system that produces double emulsions by controlling the shear stress in microchannels. The scale bar is 200 µm. Reproduced from Samandari *et al*.^53^, with the permission of ELSEVIER Publishing. (**d**) Stagnation point that divides the Droplet phase to four identical streams, each of which detaches to small droplets. The stagnation point is magnified. The scale bar is 50 μm.

Encapsulation of several particles, cells or molecules within droplets (double and multicore emulsions) has produced microtissue structures^1–3^, controlled chemical reactions^4,5^, engineered chemicals or drugs for evolving cells and enzymes^6^, and regulated cell fates^7^. A new class of multicore emulsions called multiple emulsions (MEs), is the generation of many small droplets dispersed within emulsions, themselves dispersed in a continuous phase (Fig. 1a,b)^8^. MEs have attracted many attentions playing roles as vehicles to enhance drug delivery of econazole nitrate as an antifungal targeted to deep-seated epidermal yeast infection^9^; pH-responsive cargos for effective tumor therapy to reduce the toxicity of conventional chemotherapy^10^; targeted drug delivery for the therapy of artery embolization and liver cancer^11^; and Ca-Alg/chistosan microcapsules system with embedded multivesicular liposome microparticles for diabetes treatment^12^. MEs have also demonstrated pronounced performance, such as decreasing blood cholesterol in a more effective method using encapsulated B-Sitosterol with enhanced solubility in water^13^; minimizing damage to living cells during the freezing process^14^; stabilizing coacervation process of raspberry anthocyanin known for its antioxidant activity^15^; and advanced physicochemical stabilization for therapeutic evaluation of topical hydrogels containing vitamin C-loaded self-double-emulsifying drug delivery system^16^. Other proven applications of MEs include skin infection treatment using Clotrimazole^17^; exceptional permeability of MEs atenolol self-double emulsifying drugs for gastrointestinal aqueous environment^18^; and effective digestion performance in intestinal fluids using Pickering MEs microstructures^19^.

Several different materials have been employed to constitute different phases of MEs to enhance emulsion stability and protect inner droplets against flocculation, creaming, or coalescence^20^. These materials include but not limited to liquid crystals^21–23^ and lecithin^24^; synthesized graphene oxide-polystyrene^25^; glycerol to produce polyols-in-oil-in-water^26^; natural glycyrrhizic nanofibrils assembling into a fibrillary hydrogel network to produce gelled MEs^20^; bioactive materials dispersed in glycerol with the components of glycerol and organogel matrix of sitosterol-oryzanol in sunflower oil gels to produce oleogel capsules^27^; graphene micro-aerogels embedded within soft MEs for electrochemical sensing^28^; mix of oil, toluene, water and microparticles of poly benzyl methacrylate to produce porous polystyrene monoliths MEs^29^; short-chain fatty acid within dietary fibers MEs^30^; bacterial celluloses encapsulated within protein and polyglycerol polyricinoleate MEs^31^; and eucalyptus oil, ubiquinone and fine water interfacing with hydroxy methyl cellulose and tannic acid to produce soft microcapsules of MEs^32^. Also, several emulsifiers^33^, silica nanoparticles^34–37^, colloidal materials^8,38,39^, pH stimuli-responsive polymers^40,41^, biomacromolecules^42^, surfactants^43,44^ and physical parameters^45,46^ have been incorporated to improve the MEs stability and performance^8,39,47–49^.

Conventional blending and stirring techniques are the most dominant techniques used so far for producing MEs and have been extensively used with different materials, gas or liquid phases, and media conditions to enhance MEs stability^8,36,38,39,50^. In these techniques, the shear stress was provided between two immiscible fluids to detach one of the phases and form small droplets^35,40,51^. The shear stress is exerted in the presence of colloidal materials or surfactants to prevent merging of droplets following their detachment. However, blenders and stirrers apply shear stress on the bulk of fluids that makes it cumbersome to control the shear stress distribution accurately^8,38^. The asymmetric pressure distribution limits the process control and eventuates to a variation in size for tens up to hundreds of micrometer droplets. Besides, the process is mainly a two- or multiple-step method and needs the compatibility of different emulsifiers and immiscible phases^11,22,26^.

Recently, instead of exerting shear stress using blenders or stirrers, microfluidic technology has been employed to control shear stress and produce small droplets encompassed by a Sheath (Fig. 1c)^4,47,52,53^. The shear stress is adjustable and controllable, in contrary to the conventional methods of MEs formation. The micro-scale characteristics of the flows in microchannels enable to apply a high shear stress to the flowing fluids. The shear stress is applied on a small fraction of the fluid, as several nanoliters, which makes the size of droplet precisely adjustable^54–56^ for the production of single emulsions^57,58^, multicore emulsions^59^ as well as entrapment and transportation of desired particles^60–63^, cells^64–70^ and bacteria^71^. Also, surface tension is dominant in micro-scale channels that allows to produce high-throughput droplets with identical size^72^ and shape^2,73,74^. Different structures of microchannels have been designed to produce single emulsions^75–77^, double emulsions^1,3,72,78–81^, viscous multicore emulsions^47,82–84^, and viscoelastic multicore emulsions^73,83,85^. However, generation of monodisperse MEs in microfluidics using existing techniques is yet challenging since producing many monodisperse MEs in microchannels needs several additional junctions^72,84,86^ incorporated to the fluidic design, making the actuation and control of the multiphase fluid systems very complicated. Adding several fluid junctions to the microfluidic design altogether with arduous manufacturing process intensifies pressure fluctuations within the fluid network and complicates the precise adjustment of flow rates for all fluid junctions.

In this work, we present a new microfluidic design with a new regime of droplet formation that uses fluid hydrodynamics in a single step process to harness monodisperse MEs configuration. This system can combine the advantages of producing emulsions shown in Fig. 1b,c to produce monodisperse MEs within microfluidics (Fig. 1d). It is, however, noted that this research focuses on the physics of MEs formation within microfluidics without selecting any specific type of fluids (gases or liquids) for the Sheath phase^47,48^. In the numerical simulation, a hybrid microfluidic network with two separate compartments is employed to generate MEs in microchannels: the top cross-junction compartment, and the bottom T-junction compartment. The cross-junction generates inner droplets via a flow-focusing regime while the T-junction controls the Sheath size and period of MEs formation. The device produces monodisperse MEs with identical Sheath characteristics and minimal variation in the size of micro-droplets. The new MEs formation process improves the concerned stability of existing ME generation techniques where identical Sheath size preserves MEs from merging and aggregating. The droplet formation without any emulsifier has been studied experimentally at T-junction^49,87–93^, similar to the Sheath formation process in this study. Therefore, MEs can be produced via only one emulsifier for both the Droplet and the Sheath phases to reduce the surface tension. MEs produced following the cross-junction and T-junction are finally convected to the downstream channel for further collection and manipulation. Several parameters, including three dimensionless numbers of Weber number, Reynolds number and Capillary number as well as contact angle and droplet size distribution are considered to produce rapid and reliable monodisperse MEs in a high-throughput microfluidic system.

## Results

**Figure 2:**
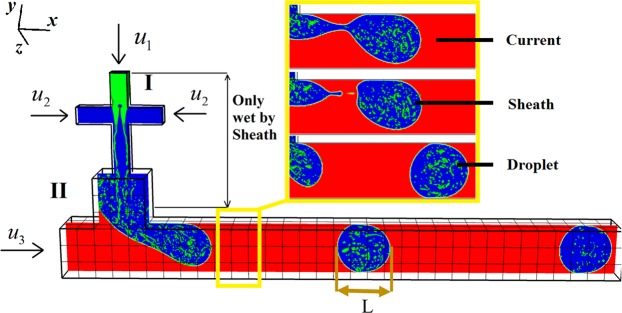
Schematic of the formation of MEs within microfluidics. The fluid flows at the inlets are shown with arrows. The inlets 1 to 3 are related to the Droplet, the Sheath, and the Current, respectively. The small droplets (green droplets) are called Droplet. The large drop or cover (blue drop) is called the Sheath. The red phase is called Current. The yellow magnified area shows three steps of the Sheath formation in the dripping regime. (top) Necking step at t = 0 (middle) and the Sheath phase detachment at t = 0.2 ms. (bottom) The generated Sheath minimizes its surface at t = 0.5 ms. The ME length is L from rear to front side of each ME.

This study focuses on the two instabilities occurring in a hybrid microfluidic network and using them to form monodisperse MEs within microfluidics. The design structure of the microfluidic system is illustrated in Fig. 2. The simulations are done in a fully-scale three-dimension (3D). Three different phases, named as Droplet, Sheath, and Current, interact with each other to control the inner droplets flowing into the Sheath. The small droplets (micro-droplets) are created with the instability provided at the cross-junction because of the presence of stagnation point exerted by the Sheath phase on the Droplet phase (Fig. 1d and Video 1). Following the formation of small droplets at the cross-junction, they move toward the T-junction and fill the forming Sheath. The MEs is the Sheath phase containing the Droplet phase inside, which gently march to the downstream microchannel (Video 2). The first instability occurs at the cross-junction, where both Droplet and Sheath phases are introduced to the inlets. Since the velocity of the Sheath phase is significant compared to the Droplet phase, a stagnation point is created at the cross-junction which splits the Droplet phase to four identical flow streams. The stagnation point moves the streams to the corners of the cross-section. The low surface tension between the Droplet and the Sheath phases hinders the droplet formation at the cross-junction and allows the Droplet phase to divide into four streams (Video 1). The stagnation point consists of two pairs of vortices (Fig. S1). These four streams march toward the expansion section placed at the joint between the cross-junction and T-junction. The sudden expansion increases the pressure of Droplet phase and decreases its velocity which intensifies both surface instability of the jet streams and Rayleigh-Plateau instability (Fig. 1d), eventuates to a new regime of droplet formation proposed in this work. Rayleigh-Plateau instability may occur for each of the jet streams even without an expansion section. However, the expansion section intensifies the instability and guarantees the droplet formation. The droplets generated from the streams have a length scale of about 10% of the cross-junction hydraulic diameter. The 14 variables selected for the three-phase flows of ME formation include three velocities (*u*_D_, *u*_S_, *u*_C_), three viscosities (*μ*_D_, *μ*_S_, *μ*_C_), three densities (*ρ*_D_, *ρ*_S_, *ρ*_C_), and three inlets hydraulic diameters (A_D_, A_S_, A_C_) for three phases as well as the two surface tensions between the Droplet and the Sheath phases (σ_D-S_) and between the Sheath and the Current phases (σ_S-C_). Given the success of dimensionless numbers in droplet microfluidics for controlling the regimes of droplet formation^3,47,73,85,94–104^, in this work, we analyze the sensitivity of the ME generating microfluidic system to each of the dimensionless numbers. Six dimensionless numbers are chosen for ME formation in our simulation to analyze the physical phenomena of ME formation. These dimensionless numbers are Weber number for the Droplet phase (We_D_), Reynolds number of the Sheath phase (Re_S_), the ratio of the Sheath net flux to the Droplet next flux (Q_S-D_), the ratio of the Current net flux to the Sheath next flux (Q_C-S_), Capillary number of the Sheath phase (Ca_S_) and Capillary number of the Current phase (Ca_C_).

We first start with one example of dimensionless numbers for simulating MEs formation is microfluidics, where these values of dimensionless numbers are selected based on the experimental data available for validating our numerical model^73^. We will then assess how alteration of each of dimensionless numbers affect the regimes of MEs formation and their stability. Therefore, dimensionless numbers are selected as ${{\rm{We}}}_{{\rm{D}}}={\rho }_{{\rm{D}}}{{u}}_{{\rm{D}}}^{2}{\rm{W}}/{\sigma }_{{\rm{D}}-{\rm{S}}}=0.625$, ${{\rm{Re}}}_{{\rm{S}}}={\rho }_{{\rm{S}}}{{u}}_{{\rm{S}}}({2}{\rm{W}})/{\mu }_{{\rm{S}}}=900$, ${{\rm{Q}}}_{{\rm{S}}-{\rm{D}}}={u}_{{\rm{S}}}{{\rm{A}}}_{{\rm{S}}}/{u}_{{\rm{D}}}{{\rm{A}}}_{{\rm{D}}}=40$, ${{\rm{Q}}}_{{\rm{C}}-{\rm{S}}}={u}_{{\rm{C}}}{{\rm{A}}}_{{\rm{C}}}/{u}_{{\rm{S}}}{{\rm{A}}}_{{\rm{S}}}=\,5$, ${{\rm{Ca}}}_{{\rm{S}}}={\mu }_{{\rm{S}}}({2}{{u}}_{{\rm{S}}})/{\sigma }_{{\rm{D}}-{\rm{S}}}=1.1$ and ${{\rm{Ca}}}_{{\rm{C}}}={\mu }_{{\rm{C}}}{{u}}_{{\rm{C}}}/{\sigma }_{{\rm{S}}-{\rm{C}}}=5\times {10}^{-3}$. Also, the desired regime of MEs formation can be controlled by altering the surface tension, viscosity ratio of phases and geometry of the microchannels. Accordingly, the velocities of the Droplet and the Sheath phases are set to 4.5 × 10^−3^ m/s and 9 × 10^−2^ m/s, respectively. The viscosity for both cases is set to 10^−5^ Pa.s and the density is set to 10^3^ kg/m^3^ for all phases. The surface tension between the Droplet and the Sheath phases is set to 1.62 × 10^−6^ N.m. The Current velocity and viscosity are set to 10^−1^ m/s and 10^−3^ Pa.s, respectively. The surface tension between the Sheath and the Current phase is set to 2 × 10^−2^ N.m. Also, both the depth and width of the cross-junction are set to 50 µm. The ME regime formed based on these selected parameters is shown in Fig. 1d.

Here, the effect of alteration in each of dimensionless numbers on formation regime of MEs is presented. Weber number for the Droplet phase (in this simulation ${{\rm{We}}}_{{\rm{D}}}={\rho }_{{\rm{D}}}{{u}}_{{\rm{D}}}^{2}{\rm{W}}/{\sigma }_{{\rm{D}}-{\rm{S}}}=0.625$) represents the balance between interfacial tension force and inertia force of the Droplet phase. The decrease in We_D_ highlights the effect of surface tension, inhibiting the split of the Droplet phase and formation of four streams at the cross-junction. This behavior is similar to the results of droplet formation in hierarchical flow-focusing microchannels, discussed somewhere else^1^. However, the effect of We_D_ on stability of the MEs needs further investigation.

The second dimensionless number is Reynolds number of the Sheath phase, (in this simulation ${{\rm{Re}}}_{{\rm{S}}}={\rho }_{{\rm{S}}}{{u}}_{{\rm{S}}}({2}W)/{\mu }_{{\rm{S}}}=900$). This value of Re_s_ intensifies the singularity, where the stagnation point occurs at the cross-junction. The stagnation point defines how the Droplet jet divides and deforms. It also shows that the effect of inertia is not negligible in MEs formation. The expansion section added to the fluidic design decreases Re_s_ and reduces the momentum of the Sheath phase, preventing its collision with the bottom wall of the main microchannel. Since the hydraulic diameter of the expansion section is three times larger than the Sheath’s inlet, the expansion section also decreases Re_s_ three times before the T-junction. Re_s_ varies between 1200 to 0.1 by changing the density of the Sheath phase from 1,333 to 0.1 kg/m^3^. This range of density covers the wide range of densities from gases like hydrogen to high viscous liquids for the Sheath phase. For higher Re_s_, the flow becomes unstable which prevents the formation of monodisperse droplets. It is worth mentioning that Re_s_ consists of the terms of Sheath velocity, density, viscosity and channel dimension. However, density of the Sheath phase is the sole parameter that can change Re_s_ without affecting other dimensionless numbers studied in this work. Therefore, altering the density of the fluid is used to determine the sensitivity of the microfluidic ME generation system to Re_s_.

**Figure 3:**
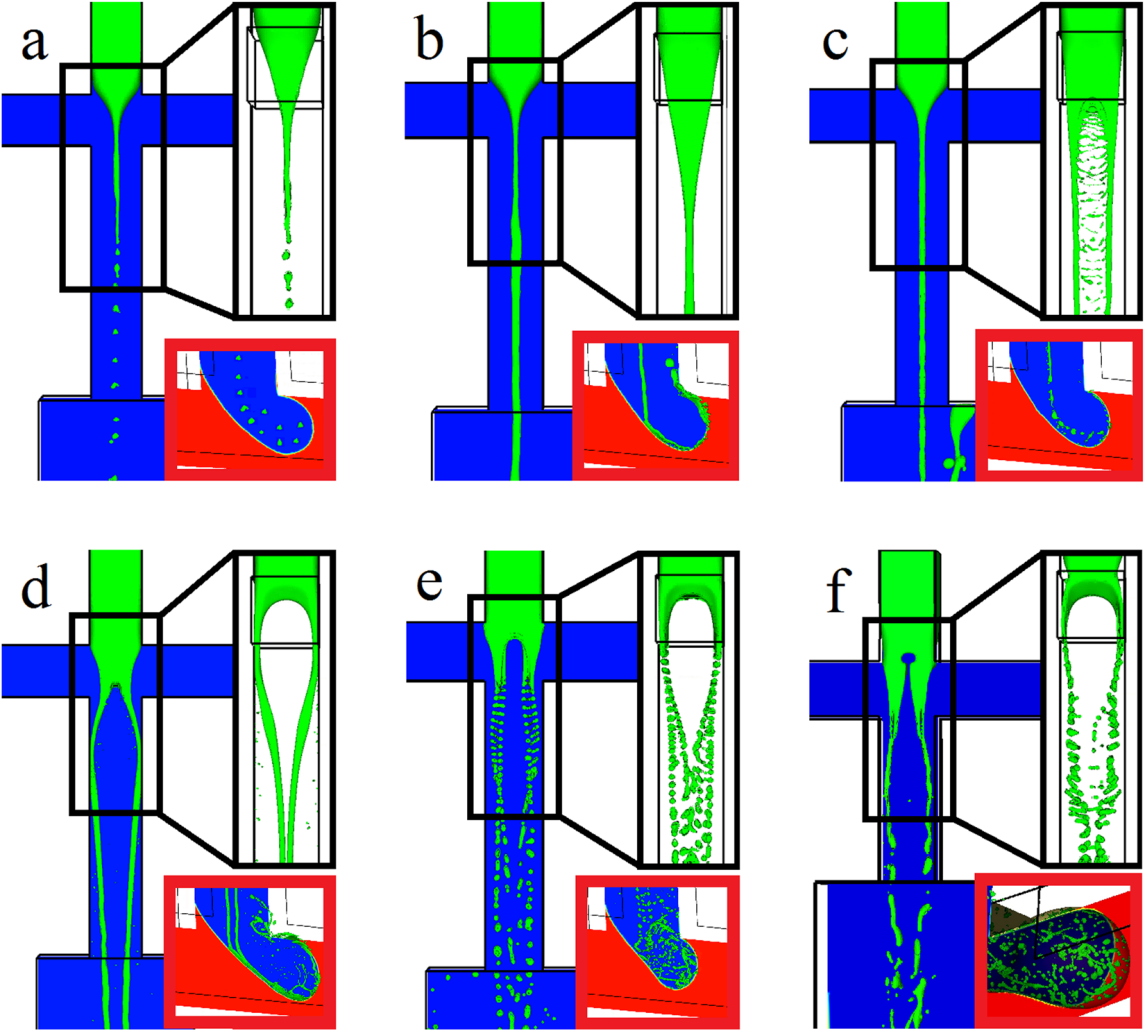
The effect of Re_s_ on the jet and droplet formation. The black magnified area, at the top right of each section, is the side view. The red magnified area, at the right bottom of each section, is the Sheath formation near T-junction. The density of the Sheath phase is altered to change Re_s_. The Sheath size is not affected by Re_s_. (**a**) Re_s_ = 0.1. (**b**) Re_s_ = 10. (**c**) Re_s_ = 50. (**d**) Re_s_ = 300. (**e**) Re_s_ = 500. (**f**) Re_s_ = 900.

Six different regimes of jet deformation during droplet formation are shown in Fig. 3. The droplet formation is mainly stimulated by Rayleigh-Plateau instability. The tip-streaming regime is dominant for Re_S_ < 0.5, where Re_s_ is not effective on jet deformation (Fig. 3a). For 1 < Re_S_ < 40, the jet elongates to the downstream channel until the Sheath phase encapsulates the jet at the T-junction (Fig. 3b). For 40 < Re_S_ < 150, the jet is divided into two streams near cross-junction because the inertia of the Sheath phase deforms the jet to a fluid sheet (Fig. 3c). For 200 < Re_S_ < 400, the inertia of the Sheath phase is strong enough to divide the Droplet phase into four similar streams (Fig. 3d). For 450 < Re_S_ < 700, the jet preserves the deformed shape and the applied shear stress from the Sheath phase on the Droplet phase is strong enough to intensify the surface instabilities and detach each of the jet streams to small droplets (Fig. 3e). For 800 < Re_S_ < 1200, the vortex near the cross-junction develops a hole inside the Droplet phase which then detaches the jet streams (Fig. 3f).

The third dimensionless number is the ratio of the Sheath net flux to the Droplet net flux, (in this simulation ${{\rm{Q}}}_{{\rm{S}}-{\rm{D}}}={u}_{{\rm{S}}}{{\rm{A}}}_{{\rm{S}}}/{u}_{{\rm{D}}}{{\rm{A}}}_{{\rm{D}}}=40$), which shows the concentration of the inner droplets in each ME. For instance, increasing the Droplet net flux increases the volume of droplets trapped inside the Sheath. Also, there are boundaries that identify the regimes of droplet formation and limit the adjustment of the net flux.

The fourth dimensionless number is the ratio of the Current net flux to the Sheath net flux, (in this simulation ${{\rm{Q}}}_{{\rm{C}}-{\rm{S}}}={u}_{{\rm{C}}}{{\rm{A}}}_{{\rm{C}}}/{u}_{{\rm{S}}}{{\rm{A}}}_{{\rm{S}}}=\,5$), which controls the Sheath size and formation characteristics. Also the fifth and sixth dimensionless numbers are the two Capillary numbers. The first Capillary number (in this simulation ${{\rm{Ca}}}_{{\rm{S}}}={\mu }_{{\rm{S}}}({2}{{u}}_{{\rm{S}}})/{\sigma }_{{\rm{D}}-{\rm{S}}}=1.1$) declares that the Sheath phase exerts the shear stress on the Droplet phase during its interaction at the cross-junction where the Sheath phase plays the role of continuous phase while the Droplet phase acts as the disperse phase. Increasing the shear stress intensifies the Rayleigh-Plateau instability and changes the size of the detached droplets and the detachment location^1^. This Capillary number can be used for adjusting the droplets size while it has a negligible effect on the Sheath size^1^. The high value of shear stress and inertia forces result in the appearance of the stagnation point at the cross-junction, making a completely different scenario of droplet formation for the Droplet phase. A high value of Ca_s_ (low surface tension σ_D-S_) along with high Re_s_ provides enough shear stress to transform the Droplet phase to four streams and create a new mechanism for the generation of the monodisperse MEs. This may equivalently infer the importance of viscosity ratio on controlling the MEs formation.

The T-junction instability is well-known and explained in detail somewhere else for five different regimes of droplet formation^75^. The dripping instability is dominant at the T-junction which can be controlled by a Capillary number via adjusting the velocity of the Current phase. The second Capillary number ${{\rm{Ca}}}_{{\rm{C}}}={\mu }_{{\rm{C}}}{{u}}_{{\rm{C}}}/{\sigma }_{{\rm{S}}-{\rm{C}}}$ is considered for the T-junction instability for controlling the Sheath size and formation characteristics. The yellow zone highlighted in Fig. 2 (shown in Video 2) show that MEs formation falls within the dripping instability regime at the T-junction where Ca_c_ = 0.005^75,83^. The results of Re_S_ > 1 show that the momentum of the Sheath phase is strong enough to make a considerable change in the flow field necessary for producing the dripping instability regime.

**Figure 4:**
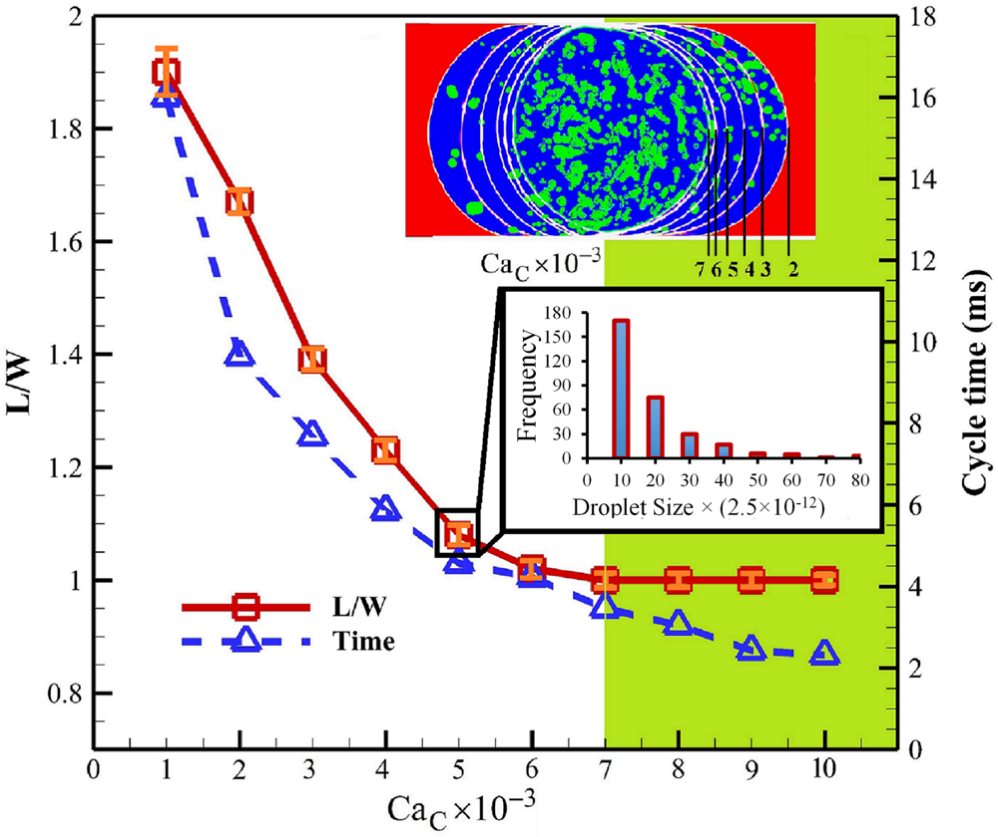
The ME formation for different values of Ca_c_. The solid line is L/W and related to the primary axis. The dashed line is the cycle time (ms) and related to the secondary axis. The size measurement and cycle time are related to the ME. The error bar for the ME size variation is shown with orange color in each simulation point. Small variation of the ME size shows its monodispersity. The black magnified area is the frequency distribution of the small droplets inside the ME against the size variation. The y axis is frequency and the x axis is number of small droplets in the range.

The velocity of the Current phase is changed to analyze the effect of Ca_c_ and determine its relative effect on the generated Sheath size and formation process (Video 3). The results show that the Sheath size increases with decreasing the velocity of the Current phase (decreasing Ca_c_) (Fig. 4). The statistical analysis of ME formation within microfluidics (Table S1 and Fig. S2) show that the size variation of MEs remains below 3% (Fig. 4). Also, the Sheath size variation among 10 MEs was measured, and the small variation in size shows the monodispersity of the Sheaths in each regime of formation (Fig. 4).

For Ca_c_ higher than 0.007, the dimensionless Sheath droplet size (L/W) remains unchanged and the generated Sheath remains spherical during its transportation through the downstream microchannel. It is because the size of the generated Sheath remains smaller than the microchannel width. The emulsion instability is also studied at the T-junction for Ca_c_simulated within the range of 0.001–0.036 with the interval of 0.001. The flow regimes are determined to be squeezing for Ca_C_ < 0.003, transition for 0.003 < Ca_C_ < 0.005, dripping for 0.005 < Ca_C_ < 0.01 and jetting for Ca_C_ > 0.01. Despite the shift of the boundaries for each regime of the T-junction instability, the sequence of ME formation regimes remains unchanged for different velocity ratio of the Current phase to the Sheath phase, also demonstrated before^91^. Unlike previous studies, the squeezing and jetting regimes for Re_s_ > 1 are not consistent and stable due to the dominance of the momentum of the Sheath phase compared to the Current phase. The inertia of the Sheath phase prevents the Current phase from smooth transporting the emerging tip at the T-junction. The mechanism underlying the Sheath generation at the T-junction in the squeezing regime is based on the pressure build-up of the Current phase at the upstream of the emerging tip. The tip blocks the Current phase following its detachment from the bottom of the main microchannel, allowing the Current phase to slightly leak from the corners of the microchannel (Fig. S3a). However, the total volume of the leakage is not enough to preserve the pressure needed for the tip transportation to the downstream microchannel. Therefore, it needs more time than usual for pressure build-up at the upstream of the tip. The characteristics of the squeezing regime are addressed in the literature^91^. For Re_s_ > 1 and Ca_C_ < 0.001, the momentum of the Sheath phase is significantly high to intensely collide the emerging tip with the bottom wall of the main microchannel (Fig. S3b,c). Afterward, the pressure drastically increases until it steers the bulk of the tip to the pinch-off location at the T-junction (Fig. S3d,e). The droplet formation is shown to be reproducible, and the tip collides the bottom wall of the microchannel in every cycle (Fig. S3f). It has already been demonstrated that the jetting regime evolves from stable to unstable with increasing Ca_c_, generating the parallel regime^75^. However, the stable jetting regime of MEs with identical Sheath sizes is observed only at 0.010 < Ca_C_ < 0.015. For Ca_c_ higher than 0.015, the generated Sheath varies in size which is no longer reliable for ME formation needed for high-throughput ME production (Video 3).

Various coating protocols, such as plasma oxidation and surface functionalizations, have been used for spatial control of contact angles within microfluidics^53,73,105^. Our ME formation microfluidic system requires partial hydrophilicity at a specific portion of the fluidic network to control the contact angle for generating stable MEs. Here in our model, the contact angle is modeled as the static contact angle, according to the model of drop shape analyzer device reported somewhere else^56^. Also, we used the validated algorithm of Afkhami & Bussmann^106,107^ for applying the contact angle in the numerical method. The given values of the contact angle are for fluids at the steady-state condition to analyze how the change of contact angle affects the droplet regime. Therefore, the effect of contact angle on ME generation within the microfluidic system is studied and shown in Fig. S4. The results show that the generated MEs under the contact angles of 0° and 20° are similar in time scale and droplet size. The cycles of droplet formation last 4.57 ms and 4.95 ms for the Current phase contact angles of 0° (180° for the Sheath phase) and 20° (160° for the Sheath phase), respectively. Accordingly, the dimensionless sizes of the Sheath phase are 1.08 and 1.17, respectively (Fig. S4). The contact angle above 10° is practical to manufacture. For the contact angle of 40°, the Sheath phase slightly attaches to the walls of the microchannel which increases the period of each cycle to 6.8 ms. The simulation shows that increasing the contact angle to above 60° inhibits the detachment of the Sheath phase at the T-junction for the formation of droplets, leading to the continues elongation of the tip to the downstream as a parallel regime (Fig. S5). The failure of droplet formation for the contact angle of above 60° is different from the results of conventional droplets formation at T-junction where the droplets form even with the contact angle of 90°^108–110^. This failure may be due to the high momentum of the Sheath phase at Re_s_ > 1 which prevents the pressure build-up for overcoming surface tension forces. The entire process for the formation of one ME lasts approximately 5 ms for We_D_ = 0.625, Re_s_ = 900, Q_s-d_ = 40, Q_c-s_ = 5, Ca_s_ = 1.1 and Cac = 0.005. The variation in droplet size at the cross-junction in the hybrid microfluidics is limited to 10% while the droplet size is very heterogeneous in previous works and varies in tens to hundreds of microns^8,38^.

## Discussion

A new configuration of multiphase flow is presented in this work to produce, for the first time, monodisperse MEs within microfluidics. Instead of using conventional methods of MEs formation that exerts shear stress on the bulk of fluids via blenders and stirrers, here we used microfluidic design to apply shear stress in a controlled fashion to a small fraction of the introduced liquid phases. The shear stress applied in micro-scale generates droplets with controllable size floating in precisely adjustable Sheath drops. This is the first attempt to produce monodisperse MEs in microchannels where two different instabilities play a significant role in high-throughput MEs formation. The essential compartments of the proposed hybrid microchannel design, investigated in this study, consist of the cross-junction, stagnation point, Rayleigh-Plateau instability, expansion section, T-junction and dripping instability. The effect of dimensionless numbers of We_D_, Q_S-D_, Q_C-S_, and Ca_S_ are qualitatively discussed based on the governing physics of MEs formation. The effects of two other dimensionless numbers of Ca_c_ and Re, in specific, are examined, and the corresponding regimes of MEs formation are quantitatively investigated. It is demonstrated that the contact angle above 60° prevents the Sheath formation. However, the contact angles less than 20° almost produce the same MEs. The quantitative effect of We_D_, Q_s-d_, Q_c-s_ and Ca_s_ needs to be further explored for future studies to reveal the boundaries and characteristics of MEs formation. It is believed that the length of the channel following the cross-junction and prior to the expansion section needs optimization in future studies. In overall, the MEs formation as a three-phase flow configuration with 14 key variables presented in this work can generate a versatile range of multiple emulsions for a variety of different applications.

## Methods

### Numerical method

The fluid flow of the three immiscible liquids within the microfluidic network is appropriately designed to attain a single-step, reliable and rapid MEs formation. The equations governing each of three phases include incompressible Navier–Stokes equation involving the surface tension term, variable-density flow pattern, and continuity equation (Eqs 1–3). The continuity equation and volume fraction are used to derive the advection equation (Eq. 4)^111^.

1 $$\rho ({{\rm{\partial }}}_{{\rm{t}}}{\boldsymbol{u}}+{\boldsymbol{u}}.{\rm{\nabla }}{\boldsymbol{u}})=-{\rm{\nabla }}p+{\rm{\nabla }}.(2\mu {\boldsymbol{D}})+\sigma \kappa {\delta }_{s}{\boldsymbol{n}},$$

2 $${{\rm{\partial }}}_{{\rm{t}}}\rho +{\rm{\nabla }}.(\rho {\boldsymbol{u}})=0,$$

3 $$\nabla .{\boldsymbol{u}}=0,$$

4 $${\partial }_{{\rm{t}}}c+\nabla .(c{\boldsymbol{u}})=0,$$

where *p* and ***u*** denote pressure and velocity vector, respectively, and ***D*** defines deformation tensor $({D}_{ij}=({\partial }_{i}{u}_{j}+{\partial }_{j}{u}_{i})/2)$. *δ*_*S*_ as the Dirac delta function and represents the presence of the surface tension coefficient (σ) on the interface. The volume fraction of each phase in every cell is defined as *c*. The dynamic viscosity and density of the fluid are defined as $\mu \equiv \mu ({\boldsymbol{x}},{\rm{t}})$ and $\rho \equiv \rho ({\boldsymbol{x}},{\rm{t}})$, respectively^112^. ***κ*** denotes the curvature radius of the interface and ***n*** defines the unit vector perpendicular to the interface^112^.

The open source code Gerris is used to simulate the multiphase flow of MEs within microfluidics. Finite volume method (FVM) discretization is used to solve numerically the governing equations^112^. The staggered temporal discretization is used for the volume fraction/density^111–113^. The volume of fluid (VOF) method is adopted to simulate the interfaces of the three immiscible fluids and the interaction of different instabilities that detach the droplets from the inlet phases. For the boundary conditions, uniform normal velocity is applied with zero gradient condition for the pressure at the inlet of the main channel (Current, *u*_3_), and at the two inlets of the cross-junction (*u*_1_ for the Droplet and *u*_2_ for the Sheath). The outlet boundary condition is set to zero pressure and zero velocity gradients. No-slip boundary condition is selected for all microchannel walls.

The multiphase interfaces are traced by a VOF function *c*(***x***, *t*). The geometrical VOF advects the volume fraction field for all the computational cells^111^. The viscosity and density are defined as Eqs (5) and (6), respectively.

5 $$\mu ({c}_{D},{c}_{C})={c}_{D}{\mu }_{D}+{c}_{C}{\mu }_{C}+(1-{c}_{C}-{c}_{D}){\mu }_{S},$$

6 $$\rho ({c}_{D},{c}_{C})={c}_{D}{\rho }_{D}+{c}_{C}{\rho }_{C}+(1-{c}_{C}-{c}_{D}){\rho }_{S},$$

where subscripts *C*, *D* and *S* represent Current, Droplet and Sheath phases, respectively^1^. The detailed description of the method is explained somewhere else^111,112^. The wetting boundary condition is illustrated in Fig. 2, where the top part of the microchannel is wetted only by the Sheath phase that means the contact angle of zero for the Sheath phase and 180° for both the Current and Droplet phases. For the T-junction bottom compartment, the wetting condition is set to zero contact angle for the Current phase and defined as non-wetting (180°) for both the Sheath and Droplet phases. Several other groups have demonstrated experimentally the wettability patterning of the microfluidic devices using spatially-controlled plasma oxidation, self-assembly and lithography techniques, which justifies the feasibility of patterning a desired contact angle within^114–117^. Also, the contact angles of 20°, 40°, 60° and 90° are simulated to analyze the effect of contact angle on MEs formation.

### Validation

**Figure 5:**
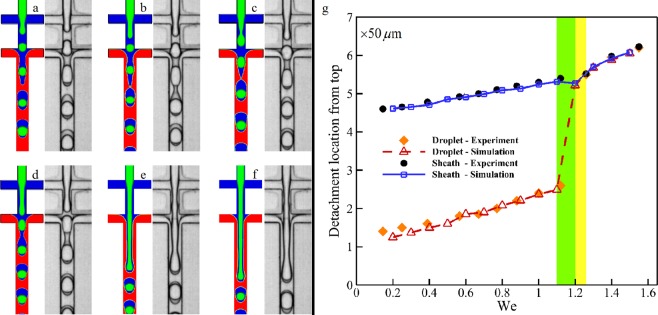
Numerical simulation compared to experimental data of Abate *et al*.^67^ (**a**) ${{\rm{W}}{\rm{e}}}_{{\rm{i}}{\rm{n}}}=\rho {u}^{2}W/\sigma =\,0.4$. (**b**) ${{\rm{We}}}_{{\rm{in}}}=0.6$. (**c**) ${{\rm{We}}}_{{\rm{in}}}=0.8$. (**d**) ${{\rm{We}}}_{{\rm{in}}}=0.9$. (**e**) ${{\rm{We}}}_{{\rm{in}}}=1.1$. (**f**) ${{\rm{We}}}_{{\rm{in}}}=1.2$. (**g**) The comparison between numerical and experimental data for the detachment location where the Droplet is formed. The highlighted area is the transition regions from two-step to one-step formation.

**Figure 6:**
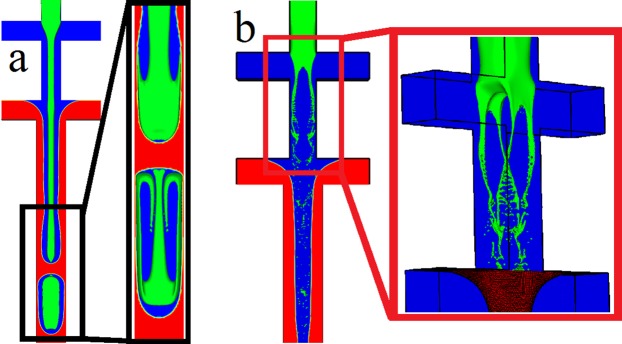
The effect of surface tension and viscosity on the formation of double emulsions. (**a**) The surface tension various from $5\times {10}^{-3}\,{\rm{N}}{\rm{.m}}$ to $1.26\times {10}^{-4}\,{\rm{N}}{\rm{.m}}$. The black magnified area is the side view. (**b**) The viscosity of the Sheath phase changes from 10^−3^ to 10^−5^ Pa.s.

The validation of the simulation results is disseminated into the dripping instability at the T-junction and Rayleigh-Plateau instability of the Droplet phase. The dripping instability at the T-junction for the formation of the Sheath is validated against the experimental data of Yeom & Lee^118^ and reported in our previous work^75^. The Rayleigh-Plateau instability of the Droplet phase arising at the expansion section is validated against the Gerris code in our previous work^111^. We previously demonstrated that the simulation of the Rayleigh-Plateau instability has a good agreement with the experimental results^1^. Also, we validated the Gerris code with the droplet formation at T-junction microchannels^75^, studied by Li *et al*.^91^ on dimensionless droplet size, and with van Steijn^119^ on velocity vectors during droplet formation step^119^. In addition, we validated the code against experimental data of Abate *et al*.^73^ (Fig. 5a–f) where Capillary number of the Sheath phase (${{\rm{Ca}}}_{{\rm{S}}}={\mu }_{{\rm{S}}}({2}{{u}}_{{\rm{S}}})/{\sigma }_{{\rm{D}} \mbox{-} {\rm{S}}}$) is set to 0.022 and Reynolds number of the Sheath phase (${{\rm{Re}}}_{{\rm{S}}}={\rho }_{{\rm{S}}}{{u}}_{{\rm{S}}}({2}W)/{\mu }_{{\rm{S}}}$) is set to 5. The numerical results are also compared to the experimental data in terms of the detachment location of the droplets where the simulations are performed for 0.2 < We < 1.5 (Fig. 5g). The transition region for experimental and simulation data are found to be 1.12 < We < 1.26 and 1.1 < We < 1.2, respectively. The green highlighted area in Fig. 5g is the transition region in simulations while both the green and yellow areas are defined as the transition regions in the experiments. The surface tension is reduced to 1.26 × 10^−4^ N.m to prevent the Droplet phase to form consecutive droplets. Therefore, the Droplet phase is elongated to the downstream channel as a jet of fluid because the surface tension is not strong enough to intensify the surface instabilities on the jet stream (Fig. 6a). Since the momentum of the Sheath phase deforms the jet stream, the viscosity of the Sheath phase is reduced from 10^−3^ to 10^−5^ Pa.s, and Re_s_ increases from 5 to 500. It is, therefore, enough to detach the Droplet phase and produce small droplets (Fig. 6b). However, the momentum of the Sheath phase is relatively strong to prevent the Sheath detachment at further downstream of the second cross-junction. An expansion section is provided after the first cross-junction to overcome the momentum of the Sheath phase and reduce Re_s_. Also, the bottom part of the microchannel is changed from cross-junction to T-junction to control the Sheath size and different regimes of the Sheath formation.

### Geometry and grid independency

**Figure 7:**
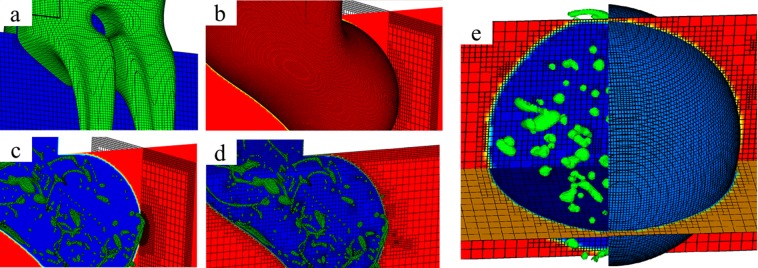
Discretization of the geometry using the AMR technique near the interfaces and the walls. The green, blue and red colors show the Droplet, the Sheath, and the Current phases, respectively. (**a**) Level 6 is used for the interface between the Droplet phase and the Sheath phase, while level 5 is used for the entire cross-junction. (**b**) Level 6 is used for the interface between the Sheath phase and the Current phase, while level 3 is used for the main channel. (**c**) Mesh structure at the y-z plane. Level 5 is used near the walls to capture the effect of shear stress. (**d**) Mesh structure at the x-y plane. (**e**) The multiple Emulsion.

Gerris uses semi-structured Quad/Octree spatial cells and adaptive mesh refinement (AMR) technique to discretize the geometry (Fig. 7a–d)^112,120^. The curvature, topology and value-based refinements are used concurrently to ensure numerical accuracy and robustness (Fig. 7e)^1,83,113,121^. The AMR technique with maximum three-level refinements is used in this work, though detailed somewhere else^83,112^. A cell of level *n* has a resolution of 2^*n*^ in each coordinate, and 0 and *n* are the refinement levels of the root cell and recursive descendant cells, respectively. The Sheath size varies less than 5% with one level increase in the superlative refinement from 5 to 6 and changes about 10% with one level increase in the superlative refinement from 4 to 5. Thus, the refinement level is set to 6 for the curvature interface (Fig. 7a,b) while the levels are set to 5 and 3 for the T-junction and the main geometry, respectively (Fig. 7c,d). The refinement level is set to 5 near the walls which ensure that the cells are refined enough to accurately predict the shear stress exerted on both the Droplet and Sheath. Level 6 for the curvature is determined to be 64 cells per 50 *μ*m, and the AMR technique executes every time step.

## Supplementary information

Video 1

Video 2

Video 3

SI

## Data Availability

The authors declare that all data supporting the findings of this study are available within the article and its Supplementary Information files or from the corresponding author upon reasonable request.
