# Supplementary material for: Rapid and Highly Controlled Generation of Monodisperse Multiple Emulsions via a One-Step Hybrid Microfluidic Device
Source: Sci Rep. 2019 Sep 3;9:12694. doi: 10.1038/s41598-019-49136-7 (PMC6722102; doi:10.1038/s41598-019-49136-7)
Supplement: Supplementary file 4 — SI [file 41598_2019_49136_MOESM4_ESM.docx]

**Rapid and Highly Controlled Generation of Monodisperse Multiple Emulsions via a One-Step Hybrid Microfluidic Device**

Milad Azarmanesh**[[1]](#footnote-1)**^1^**,** Saleh Bawazeer^1^**,** Abdulmajeed A. Mohamad*^1^**,** Amir Sanati-Nezhad*^1,2^

*^1^Department of Mechanical and Manufacturing Engineering, University of Calgary, Calgary, Alberta T2N 1N4, Canada*

*^2^Center for Bioengineering Research and Education, Biomedical Engineering Program, University of Calgary, Calgary, Alberta T2N 1N4, Canada*

* Corresponding authors

E-mails: [mohamad@ucalgary.ca](mailto:mohamad@ucalgary.ca) , [amir.sanatinezhad@ucalgary.ca](mailto:amir.sanatinezhad@ucalgary.ca)

**Supplementary Information (SI)**

Addition figures of numerical simulations are provided in this section for better understanding of the mechanisms of multiple emulsion formation within microfluidic.

| (a) | (b) | | (c) |
| --- | --- | --- | --- |
| 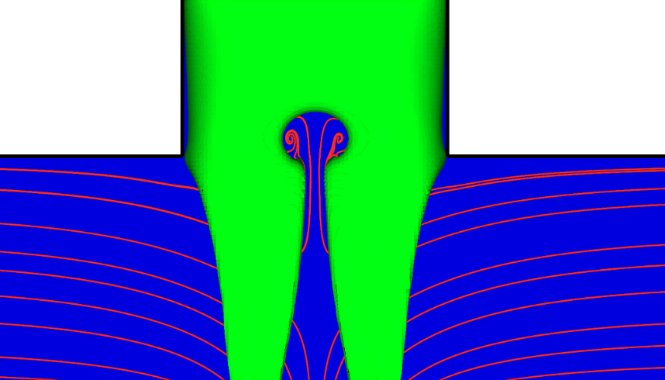 | 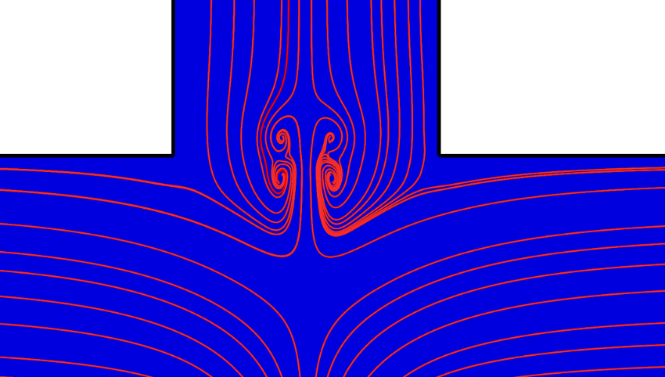 | | 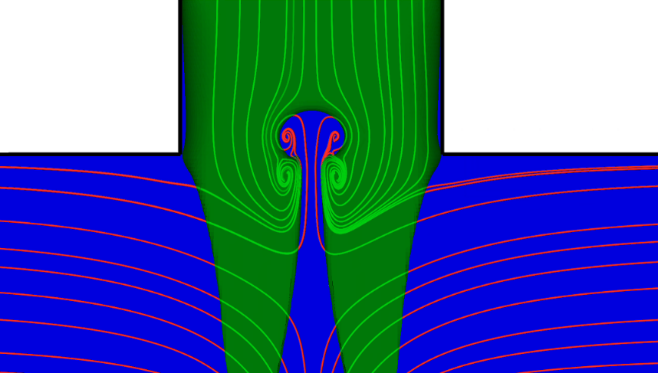 |
| (d) | | (e) | |
| 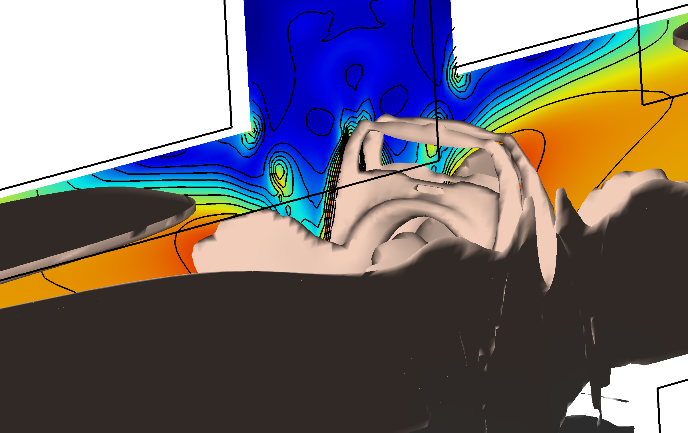 | | 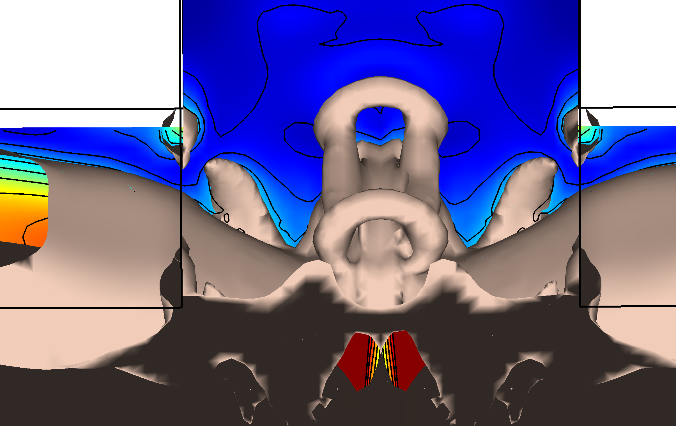 | |

**Figure S1**. Stagnation point and four vortices for the conditions with $\text{We}_{\text{D}}=\rho_{\text{D}}u_{\text{D}}^{2}\text{W}/\sigma_{\text{D} -\text{S} }= 0.625$ $\text{Re}_{\text{S}}=\rho_{\text{S}}u_{\text{S}}\text{(}2\text{W)}/\mu_{\text{S}}= 900$, $\text{Q}_{\text{S-D}}=u_{\text{S}}\text{A}_{\text{S}}/u_{\text{D}}\text{A}_{\text{D}}= 40$, $\text{Q}_{\text{C-S}}=u_{\text{C}}\text{A}_{\text{C}}/u_{\text{S}}\text{A}_{\text{S}}= 5$ and $\text{Ca}_{\text{S}}=\mu_{\text{S}}(2u_{\text{S}})/\sigma_{\text{D} \text{-} \text{S} }= 1.1$. (a) One pair of vortices is apparent and the other one is hidden under the jet flow of the Droplet phase. (b) Two pairs of vortices are visible, and the jet flow is not shown. (c) Transparent view of (a) and (b). (d) The iso-surface for the vortices (the side view). (e) The iso surfaces for the vortices (the front view).

| (a) | (b) |
| --- | --- |
| 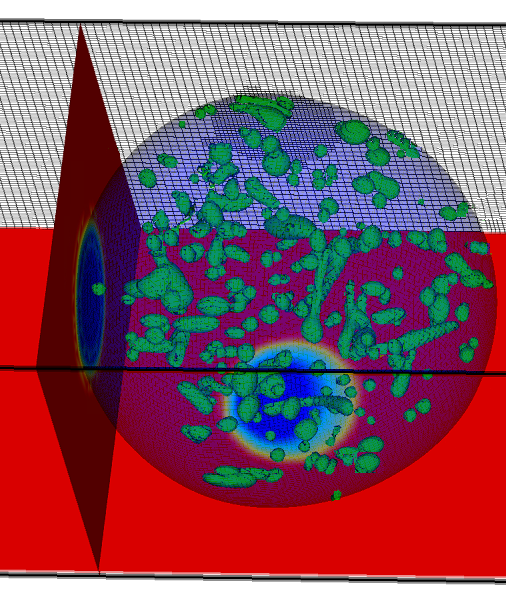 | 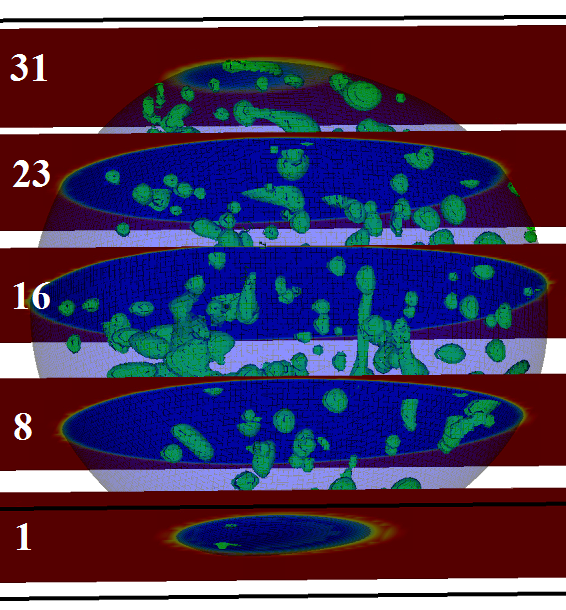 |

**Figure S2**. A sample ME formed after T-junction (shown in Figure 2). (a) The 3D view of the ME cut with y-z plane. (b) Example slices of the ME to measure the area covered by the droplets and determine the size distribution of the small droplets. In total, 307 areas (307 droplet cross-section) were analyzed to measure the size distribution of small droplets inside the ME.

| (a) | (b) |
| --- | --- |
| 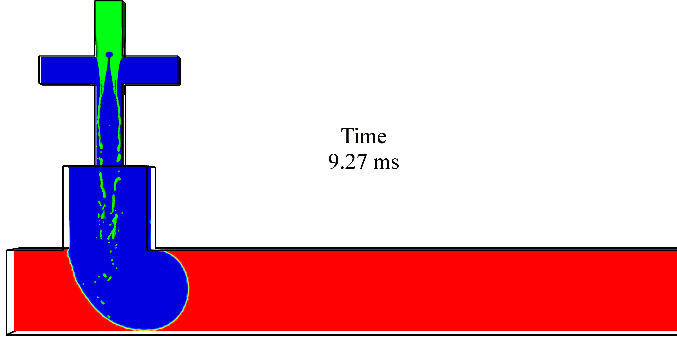 | 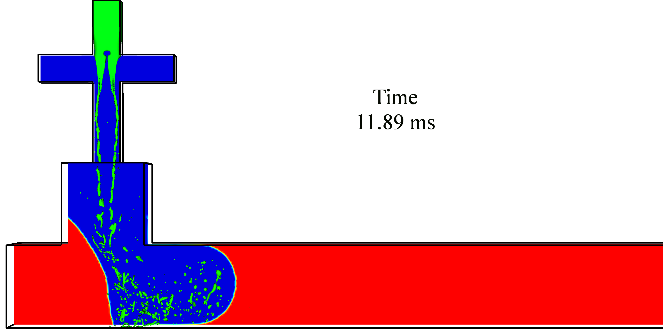 |
| (c) | (d) |
| 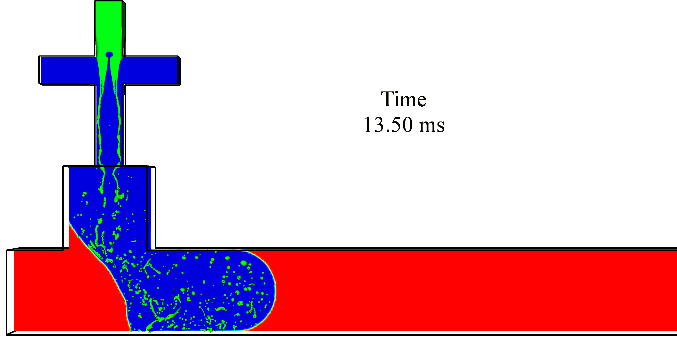 | 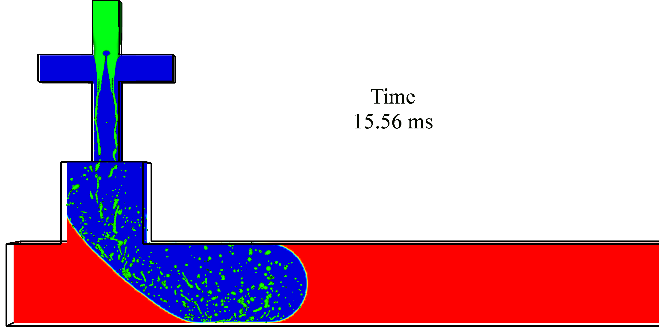 |
| (e) | (f) |
| 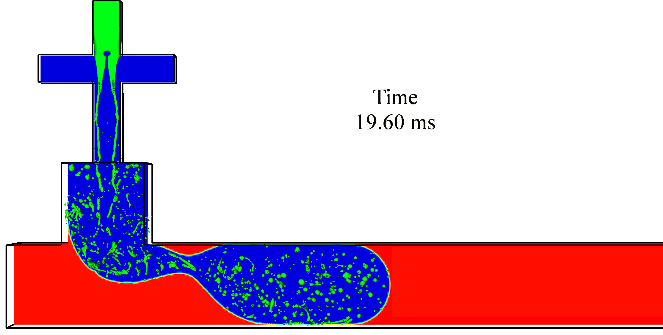 | 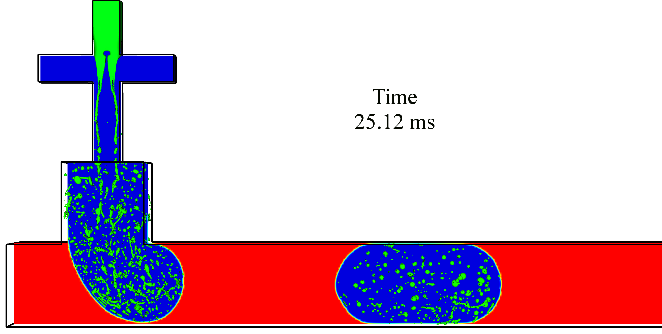 |

**Figure S3**. Droplet formation in squeezing regime when the Sheath phase impacts the bottom of the microchannel. (a) before the impact at 9.27 ms. (b) after the first impact at 11.89 ms. The pressure at the back of the tip is growing because the flow of the Current phase only leaks from corners. (c) The Current phase pushes the Sheath phase to the downstream microchannel at 13.5 ms. (d) The Sheath curvature is similar to other squeezing regimes of droplet formation at 15.56 ms. (e) Pinch-off location, one step before the detachment at 19.6 ms. (f) The final step of ME formation at 25.15 ms.

| (a) | (b) | (c) |
| --- | --- | --- |
| 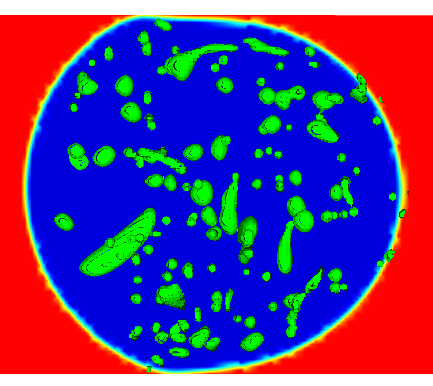 | 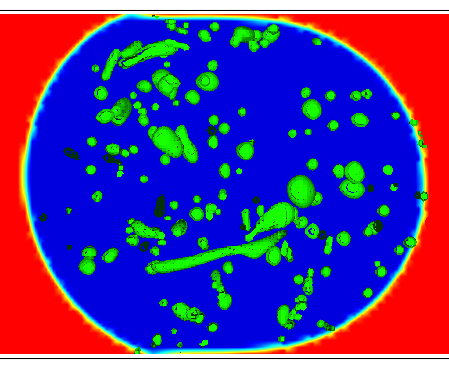 | 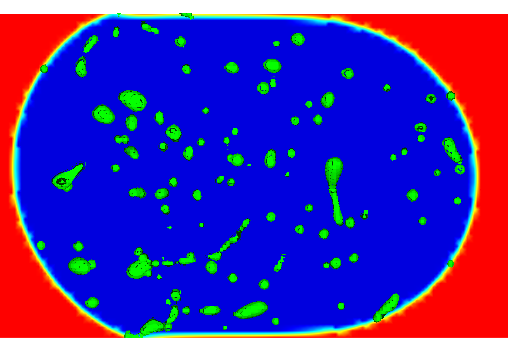 |
| 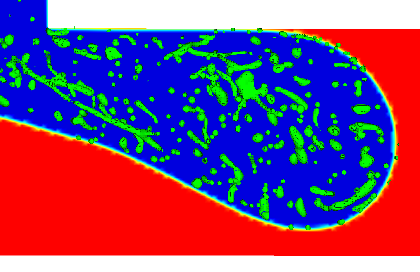 | 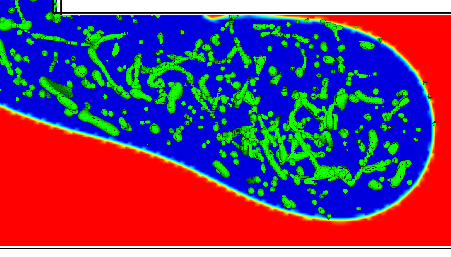 | 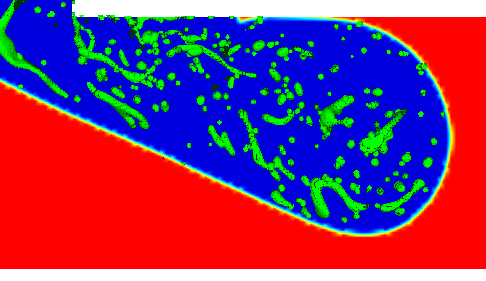 |

**Figure S4**. Different contact angles used for the Current phase (varies between zero to 40^o^). (a) The contact angle of 0^o^ for the Current phase and 180^o^ for the Sheath phase. (b) The contact angle of 20^o^ for the Current phase and 160^o^ for the Sheath phase. (c) The contact angle of 40^o^ for the Current phase and 140^o^ for the Sheath phase.

| (a) | 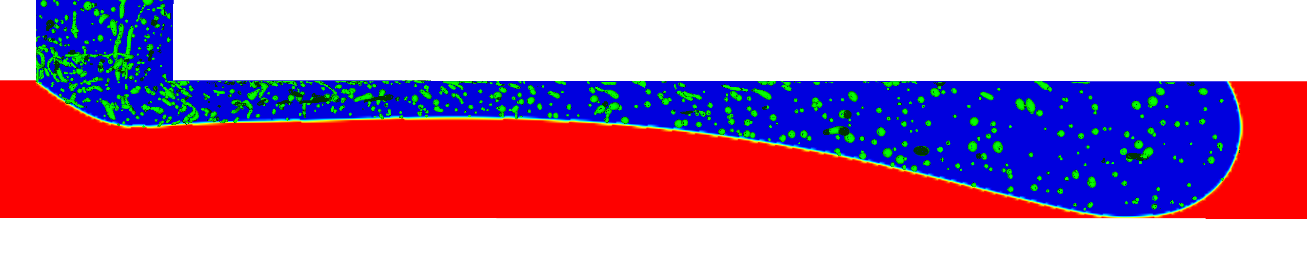 |
| --- | --- |
| (b) | 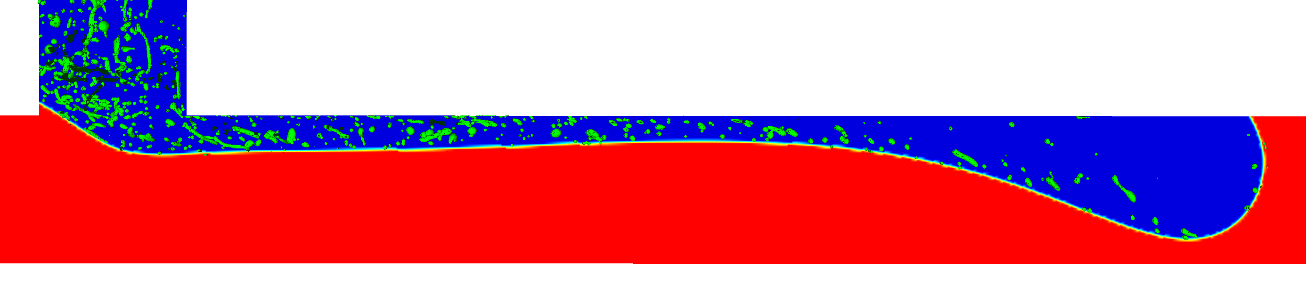 |

**Figure S5**. Different contact angles used for the Current phase (varies between 60^o^ to 90^o^). (a) The contact angle of 60^o^ for the Current phase and 120^o^ for the Sheath phase. (b) The contact angle of 90^o^ for the Current phase and 90^o^ for the Sheath phase.

**Table S1.** Size distribution of the small droplets inside the MEs right after the T-junction (shown in Figure 2). The graphic presentation of the data in this table is illustrated in black magnified area in Figure 4. In total, 307 cross-section area for the small droplets were measured inside MEs (shown in Figure S3b).

| Range of droplet size * 2.5 ${[\mu m]}^{2}$ | Frequency (number of droplets in the range) |
| --- | --- |
| 0 | 0 |
| 0-5 | 107 |
| 5-10 | 63 |
| 10-15 | 39 |
| 15-20 | 36 |
| 20-25 | 20 |
| 25-30 | 10 |
| 30-35 | 9 |
| 35-40 | 8 |
| 40-45 | 4 |
| 45-50 | 2 |
| 50-55 | 3 |
| 55-60 | 2 |
| 60-65 | 0 |
| 65-70 | 1 |
| 70-75 | 2 |
| 75-80 | 1 |

| Slice number | Droplet size * 2.5 ${[\mu m]}^{2}$ | | | | | | | | | | | | | | | | | | |
| --- | --- | --- | --- | --- | --- | --- | --- | --- | --- | --- | --- | --- | --- | --- | --- | --- | --- | --- | --- |
| 1 | - |  |  |  |  |  |  |  |  |  |  |  |  |  |  |  |  |  |  |
| 2 | 11 | 23 |  |  |  |  |  | Average area = 13.403 | | | | | | | | |  |  |  |
| 3 | 8 | 11 | 6 | 31 | 37 |  |  | Standard Deviation = 13.410 | | | | | | | | |  |  |  |
| 4 | 6 | 3 | 22 |  |  |  |  | Cumulative = 4,115 | | | | | | | | |  |  |  |
| 5 | 19 | 26 | 12 | 20 |  |  |  |  |  |  |  |  |  |  |  |  |  |  |  |
| 6 | 6 | 5 | 8 | 15 |  |  |  |  |  |  |  |  |  |  |  |  |  |  |  |
| 7 | 8 | 8 | 6 | 26 | 15 |  |  |  |  |  |  |  |  |  |  |  |  |  |  |
| 8 | 4 | 5 | 17 | 8 | 6 | 20 | 18 | 10 | 5 | 10 | 23 | 37 |  |  |  |  |  |  |  |
| 9 | 3 | 3 | 16 | 4 | 9 | 13 | 25 | 3 | 15 | 8 | 7 | 36 | 5 | 13 | 5 | 5 | 6 |  |  |
| 10 | 31 | 17 | 14 | 20 | 8 | 25 | 12 | 4 | 7 | 16 | 12 | 36 | 9 | 4 | 6 | 4 | 5 |  |  |
| 11 | 44 | 11 | 17 | 14 | 29 | 12 | 9 | 16 | 5 | 26 | 4 | 10 |  |  |  |  |  |  |  |
| 12 | 11 | 3 | 3 | 3 | 6 | 10 | 34 | 3 | 3 | 39 | 2 | 26 |  |  |  |  |  |  |  |
| 13 | 16 | 9 | 4 | 5 | 5 | 27 | 71 | 7 | 34 | 2 | 15 | 15 | 7 | 22 | 34 | 11 |  |  |  |
| 14 | 68 | 17 | 13 | 6 | 10 | 14 | 16 | 16 | 25 | 16 | 7 | 53 | 6 | 49 |  |  |  |  |  |
| 15 | 2 | 42 | 14 | 8 | 3 | 17 | 2 | 5 | 1 | 30 | 12 | 52 | 4 | 6 | 3 | 1 | 20 | 4 |  |
| 16 | 1 | 6 | 4 | 18 | 3 | 4 | 19 | 9 | 12 | 16 | 17 | 13 | 10 | 7 | 22 | 11 | 2 | 1 | 71 |
| 17 | 2 | 24 | 4 | 12 | 4 | 3 | 3 | 2 | 1 | 42 | 2 | 10 | 11 | 29 | 23 |  |  |  |  |
| 18 | 1 | 3 | 1 | 24 | 25 | 20 | 22 | 17 | 5 | 10 | 47 | 27 | 16 | 2 | 1 | 18 | 33 |  |  |
| 19 | 4 | 5 | 14 | 6 | 1 | 1 | 1 | 3 | 23 | 19 | 7 | 39 | 19 | 5 | 5 | 6 | 9 | 10 | 42 |
| 20 | 4 | 7 | 3 | 18 | 2 | 9 | 6 | 1 |  |  |  |  |  |  |  |  |  |  |  |
| 21 | 1 | 6 | 2 | 1 | 19 | 5 | 12 | 8 | 1 | 23 | 17 | 3 | 12 |  |  |  |  |  |  |
| 22 | 5 | 1 | 1 | 12 | 9 | 3 | 23 | 7 | 13 | 3 | 10 | 4 | 4 |  |  |  |  |  |  |
| 23 | 2 | 1 | 32 | 9 | 7 | 5 | 3 | 1 | 22 | 10 | 5 |  |  |  |  |  |  |  |  |
| 24 | 2 | 10 | 3 | 5 | 1 | 1 | 59 | 15 | 76 | 16 | 19 | 4 | 3 |  |  |  |  |  |  |
| 25 | 1 | 1 | 15 | 38 | 28 | 4 | 25 | 1 | 60 | 6 | 19 | 4 |  |  |  |  |  |  |  |
| 26 | 7 | 23 | 2 | 22 | 11 | 1 | 1 | 10 | 5 |  |  |  |  |  |  |  |  |  |  |
| 27 | 16 | 37 | 7 | 20 | 15 |  |  |  |  |  |  |  |  |  |  |  |  |  |  |
| 28 | 5 | 8 | 32 | 11 | 8 | 12 | 35 |  |  |  |  |  |  |  |  |  |  |  |  |
| 29 | 9 | 1 | 1 | 5 | 15 | 53 |  |  |  |  |  |  |  |  |  |  |  |  |  |
| 30 | 1 | 2 | 10 | 31 |  |  |  |  |  |  |  |  |  |  |  |  |  |  |  |
| 31 | - |  |  |  |  |  |  |  |  |  |  |  |  |  |  |  |  |  |  |

1. [↑](#footnote-ref-1)
